# Implications of MicroRNAs in Oncolytic Virotherapy

**DOI:** 10.3389/fonc.2017.00142

**Published:** 2017-07-04

**Authors:** Xavier Bofill-De Ros, Maria Rovira-Rigau, Cristina Fillat

**Affiliations:** ^1^Institut d’Investigacions Biomèdiques August Pi i Sunyer (IDIBAPS), Barcelona, Spain; ^2^Centro de Investigación Biomédica en Red de Enfermedades Raras (CIBERER), Barcelona, Spain

**Keywords:** oncolytic viruses, microRNA, gene regulation, detargeting, host–virus interaction

## Abstract

MicroRNAs (miRNAs) are an abundant class of small non-coding RNA molecules (~22 nt) that can repress gene expression. Deregulation of certain miRNAs is widely recognized as a robust biomarker for many neoplasms, as well as an important player in tumorigenesis and the establishment of tumoral microenvironments. The downregulation of specific miRNAs in tumors has been exploited as a mechanism to provide selectivity to oncolytic viruses or gene-based therapies. miRNA response elements recognizing miRNAs expressed in specific tissues, but downregulated in tumors, have been inserted into the 3′UTR of viral genes to promote the degradation of these viral mRNAs in healthy tissue, but not in tumor cells. Consequently, oncolytic virotherapy-associated toxicities were diminished, while therapeutic activity in tumor cells was preserved. However, viral infections themselves can modulate the miRNome of the host cell, and such miRNA changes under infection impact the normal viral lifecycle. Thus, there is a miRNA-mediated interplay between virus and host cell, affecting both viral and cellular activities. Moreover, the outcome of such interactions may be cell type or condition specific, suggesting that the impact on normal and tumoral cells may differ. Here, we provide an insight into the latest developments in miRNA-based viral engineering for cancer therapy, following the most recent discoveries in miRNA biology. Furthermore, we report on the relevance of miRNAs in virus–host cell interaction, and how such knowledge can be exploited to improve the control of viral activity in tumor cells.

## Introduction

MicroRNAs (miRNAs) are small non-coding RNA molecules (~22 nt) that can negatively regulate the expression of large networks of genes (1). Not surprisingly, miRNA dysregulation impacts virtually all cancer-related processes (proliferation, cell death, migration, and cell cycle, among many others). Such dysregulation provides clear hallmark miRNA signatures that can distinguish between normal cells and the tumor cells of many different types of malignancy (1).

In this regard, therapeutic strategies rely either on the reintroduction of the individual miRNAs involved in tumor suppression functions, such as miR-34 (2), or on reducing oncogenic miRNAs with antisense oligonucleotides—“antagomirs” (3). Interestingly, Brown and coworkers exploited the differences in miRNA expression between tissues and proposed a novel mechanism to control transgene expression, based on the differential expression of miR-142 among lineages of hematopoietic cells (4). Selectivity was achieved by the introduction of engineered target sites, or miRNA response elements (MREs). Later, oncolytic virotherapy also incorporated MREs to control the expression of viral or suicide genes. MREs can attenuate oncolytic viruses in non-tumoral tissue and therefore avoid the undesired toxicity associated with viral tropism when administered systemically or locoregionally (5–8). Posttranscriptional targeting with MREs could be complemented with transcriptional or transductional targeting to enhance the selectivity of oncolytic viruses.

The study of viral miRNAs and host responses has also shown the relevance of miRNAs in the regulation of viral replication. There are, in fact, several examples of how viral infection modulates cellular miRNome, with consequences on both viral activity and host cell functionality.

This review discusses the latest developments with respect to fine-tuning oncolytic viruses, based on the viral engineering of MREs, and looks at the functional consequences of the interplay between miRNAs and viruses.

## The Mechanism of Action of miRNAs

Most miRNAs are transcribed from miRNA genes and follow a canonical miRNA biogenesis pathway. miRNAs are transcribed to primary miRNA transcripts (pri-miRNAs) that are processed by the RNase III enzyme Drosha in the nucleus to generate precursor miRNA that are exported by Exportin-5 to the cytoplasm. There they are recognized and cleaved by another RNase III enzyme, Dicer, to give rise to ~22 nt miRNA duplexes. They are then loaded onto the RISC complex, where the Ago proteins will help with the unwinding of the miRNA duplexes to form a functional miRNA-induced silencing complex that will recognize target mRNAs and interfere with their expression. Target mRNA recognition will be based on partial complementarity of miRNA sequences and the 3′UTR of the mRNAs (9).

miRNAs modulate gene expression through mechanisms of translational inhibition, mostly at the initiation step, and mRNA destabilization as a consequence of mRNA deadenylation and mRNA decay. These mechanisms may occur sequentially, with mRNA destabilization as the dominant effect. In consequence, the repression of miRNAs target genes can be evidenced by depletion of the mRNA content (10, 11).

## Controlling Viral Replication through MREs

The selectivity of oncolytic viruses can be determined through the introduction of MREs, preferentially in the 3′UTR of viral genes (12). Noticeably, MREs can be inserted into virtually any viral mRNA. Comparable efficiencies have been observed with MREs targeting early phase transcription factors or late phase capsid structural proteins. Examples of the elements targeted are ICP4, ICP27, and glycoprotein H in herpes simplex virus (13–15), E1A and L5 (fiber) in adenovirus (15, 16), or M and L in vesicular stomatitis virus (17, 18). Interestingly, targeting early phase proteins reduces toxicity derived from its own expression and that of downstream genes, offering a greater safety margin than when targeting late phase proteins (16). However, the use of MREs to target both genomic and messenger RNA in RNA viruses showed efficient repression of mRNAs only. The efficacy of miRNA repression in genomic RNAs is reduced due to secondary structures and scaffold proteins that protect the viral genome (5, 19, 20).

The base pairing of the miRNA and target genes usually displays partial complementarity, restricted to nucleotides 2–7 of the miRNA, and known as the “seed” sequence (21, 22). However, partial complementarity can only mediate translational repression and mRNA decay (10, 23–25). To achieve a fast and robust effect on the control of viral replication, MREs must be designed to trigger direct cleavage in the viral mRNA. Although all human Argonaute proteins (Ago1–4) are capable of promoting a translational repression pathway, only Ago2 has endonucleolytic activity (26, 27). In order to trigger Ago2-mediated direct cleavage, the base-paring miRNA:MRE between nucleotides 10 and 11 must be complete (23). This mechanism of action exploits the same mechanisms as RNAi silencing (shRNA and siRNA) (28). The factors that can influence the efficacy of the MRE include the expression levels and profiles of Ago proteins between tissues (29) and the modulation of their activity by covalent modification (30, 31).

**Figure 1:**
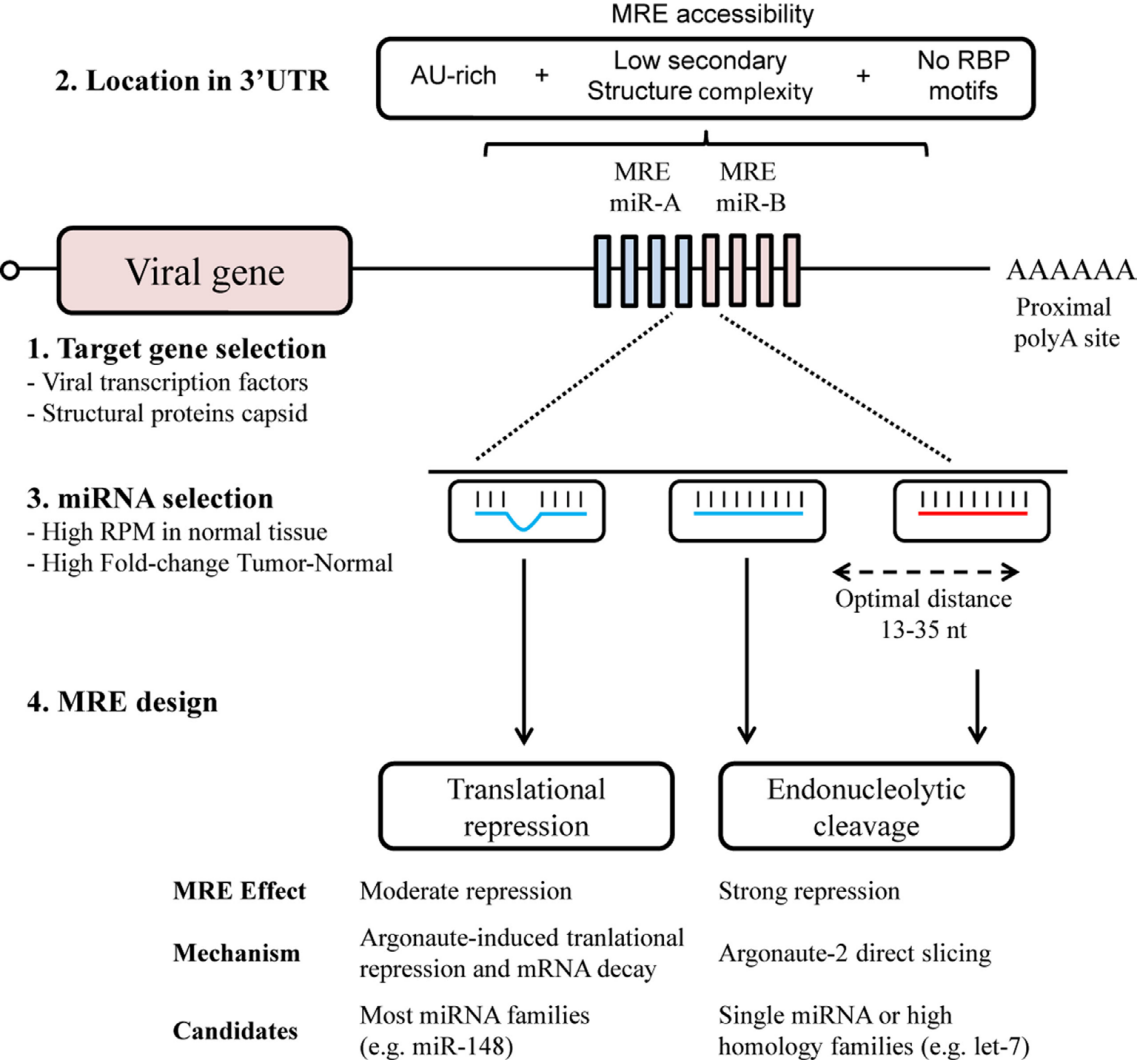
MicroRNA response element (MRE) design for an optimal selectivity of oncolytic viruses. The design of MRE in oncolytic virotherapy has to take into account several elements regarding the biology of the virus and the microRNA (miRNA) regulatory pathways. Although all viral genes with 3′UTR are susceptible to be targeted though MRE (1), it is of notice the toxicity of some viral proteins. An adequate location in the 3′UTR (2) together with a sufficient miRNA expression (3) will contribute to the target recognition. Perfect miRNA–MRE complementarity will trigger Ago2 slicing activity (4); however, imperfect base-paring with other miRNA family members will also contribute to the regulation though translational repression.

Elements of the design of MRE that contribute to its efficacy include the number of target sites, the distance between target sites, the sequence composition and local RNA structure surrounding its location in the 3′UTR (Figure 1). The optimal design for MRE should include a range of target sites, usually from 2 to 8, allowing a dose-dependent response to miRNA concentration (32), and a seed separation of between 13 and 35 nt to avoid steric hindrance (33). Access to target sites can usually be estimated through the secondary structure, with the minimum free energy (mfe) and AU-richness surrounding the site (34–36). Moreover, sites for RNA-binding proteins should be avoided since they may also mask MREs and hamper recognition. Hence MRE engineering might benefit from a computed selection of spacers, to promote optimal separation and secondary structure.

The miRNAs considered as regulators to bind to MREs are selected from those that present abundant expression in normal tissue, but a significantly decreased expression in tumors, such as the ubiquitously expressed miRNA let-7 family (18, 37, 38). Other studies have exploited tissue-specific miRNAs. Some examples of tissue-specific MREs are miR-122 for liver (6, 7), miR-7 for brain (39), miR-148a for pancreas (39, 40), and miR-192 for heart (41). In this regard, miRNA belonging to the same family can be used to increase the efficacy of MRE. The extensive homology presented by miRNA of the same family allows sub-optimal recognition of the MRE (Figure 1). An increased level of complexity in the selection of miRNA candidates arises with the design of MREs for multiple organs detargeting, usually non-tumoral cells surrounding the tumor and tissues with native viral tropism (42). This can be achieved by using miRNAs present in both organs, or by combining multiple miRNAs (40). In this context, the extensive miRNome data in The Cancer Genome Atlas (https://cancergenome.nih.gov/) for 33 types of cancer and normal tissue constitutes an invaluable resource for the selection of candidate miRNAs (43). Of special consideration when seeking to fine-tune oncolytic virus activity is the diversity of cell types in tissue, especially in approaches using locoregional administration. Here, MREs could also provide the desired level of selectivity, for example, miR-375 has been described almost exclusively in the beta and alpha cells present in pancreatic islets (44, 45).

An aspect of MRE design, aside from selectivity, that has yet to be tested for oncolytic viruses, is the incorporation of MREs as miRNA sponges or decoys. In other areas of gene therapy, sponge MREs have been used to reduce the effective amount of miRNA content (46–48). These particular MRE designs are characterized by a bulge that impairs the slicing activity of Ago2, while promoting miRNA degradation by way of trimming and tailoring (49). Sponge MREs could be incorporated into oncolytic viruses to downregulate the miRNAs involved in tumor progression (50), increase viral replication, or attenuate host antiviral response (51).

## Host miRNAs Response to Viral Infection

When a virus infects a cell, a host–virus relationship is established, creating an intricate network of interactions characterized by the massive reprogramming of cellular gene expression. The expression profile of cellular mRNAs and miRNAs is affected during viral infection (52, 53). Changes in the host miRNA profile have been reported after infection with adenoviruses (54–56), influenza viruses (57, 58), HIV-1 (59), Epstein–Barr virus (EBV) (60, 61), human cytomegalovirus (HCMV) (62), human herpes virus 1 (HSV-1) (63), and respiratory syncytial virus (64).

By merely observing miRNA profile change, it is difficult to discern whether miRNA deregulation is the consequence of a host-immune response to the infection or if it is triggered by the virus to favor replication. Comparative studies of the expression profiles of different viral infections and the analysis of miRNA targets can help elucidate the significance of deregulation.

**Figure 2:**
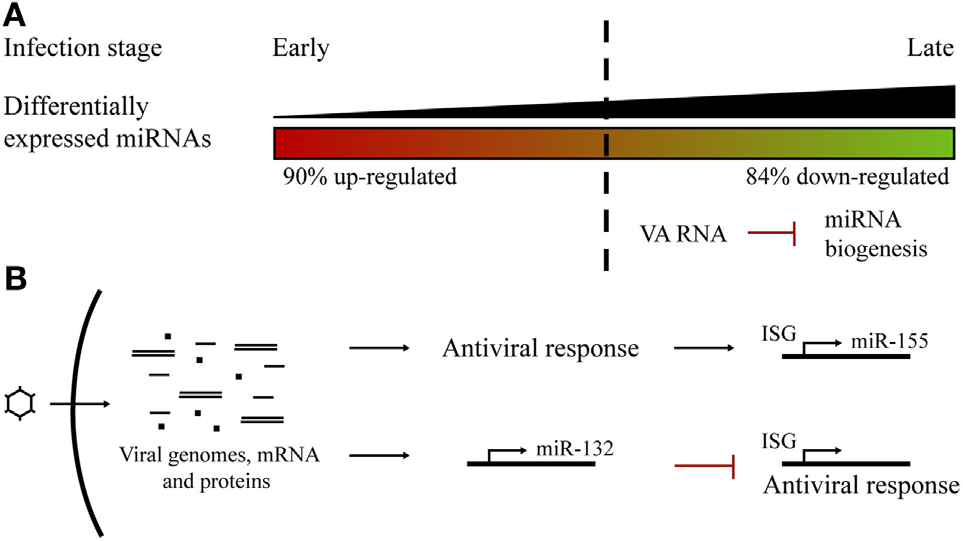
Host microRNA (miRNA) profile alterations in adenoviral infections. **(A)** Changes in the miRNA profile during the course of an adenoviral infection. There was a switch from more upregulated miRNAs at early steps to more downregulated at late phases, probably as a consequence of VA RNAs competition with host miRNA biogenesis. **(B)** Adenoviral infections trigger the overexpression of specific miRNAs. Host miR-155 expression is induced as a consequence of the cell antiviral response. Viral infection promotes the expression of miR-132 to counteract the antiviral interferon response.

The miRNA profile after adenoviral infection has been studied for adenovirus type 3 in human laryngeal epithelial cells (56), adenovirus type 2 (Ad2) in human primary cells (54), and, more recently, for adenovirus type 5 (Ad5) in prostate cancer cells (55). Ad2 infection studies showed that a correlation between the progression of the infectious cycle and the level of miRNA deregulation could be established. Changes in the profile extend from more upregulated, during the early stages of infection, to more downregulated miRNAs at the later phases (54). Massive miRNA downregulation could be the consequence of the expression of the VA (viral associated) RNAs codified by the virus at the later stages of the infection, competing with endogenous miRNA biogenesis (55) (Figure 2A). In fact, the same phenomenon was observed following Ad5 infection (55). Ad2 and Ad5 infection triggered miR-155 upregulation, an effect also observed after VSV (65) and EBV infections (61), suggesting that miR-155 could act as a host antiviral miRNA. It is also known that miR-155 is induced in macrophages, in response to interferon pathway activation (66).

Another miRNA that has been reported to be upregulated in cells infected by several viruses is miR-132. It has been found to overexpress after adenovirus (54, 55), HSV, KSHV, and HCMV infection (67). In contrast to miR-155, miR-132 acts by limiting host antiviral response since it exerts a negative effect on the expression of interferon-stimulated genes. This is a viral strategy which seeks to evade host antiviral response and promote viral replication (67, 68) (Figure 2B).

It is probable that viruses have evolved to induce the downregulation of interference miRNAs and favor the upregulation of miRNAs that can facilitate viral replication (54) (Figure 2B). Several examples illustrate this view. HSV-1 causes a series of changes in the miRNA profile and antagonizes host defenses by inducing miR-23a and miR-649 expression. These miRNAs, respectively, target *IRF1* and *MALT1* genes, involved in the antiviral signaling pathway (63, 69). In turn, the HSV-1 ICP4 protein induces the expression of miR-101, which limits virus replication to ensure the survival of host cells and therefore support persistent HSV-1 infection (70). In cells infected with Reolysin, a reovirus currently being tested for the treatment of several cancers (71), Nuovo et al. observed a modulation of certain miRNAs, with clear downregulation of let-7d, facilitating the productive viral infection and apoptosis-related death of the cancer cells (72). Hepatitis C virus (HCV) inhibits type I IFN production by upregulating the expression of miR-21 (73), while influenza virus activates the expression of miR-485, which targets the cytosolic sensor of viral RNA RIG-1 (57), and HIV-1 actively suppresses the expression of miR-17 and miR-20a that act against the virus (74).

Regardless of the miRNA changes triggered by viral infection, most cells are already equipped with miRNAs that will interact with viral genes. Antiviral miRNAs, such as miR-24 and miR-93, have been described to inhibit viral replication by directly targeting viral genes. Otsuka and coworkers described miRNA targeting VSV L and P protein genes, therefore inhibiting VSV replication (75). Such is also the case for cellular miR-32, which targets a sequence in the genome of primate foamy virus type 1 (76), or host miR-214, which is capable of inhibiting adenovirus replication by targeting the 3′UTR of E1A mRNA (77). By contrast, there are pro-viral miRNAs, such as miR-122, highly expressed in the liver, which interacts with the HCV genome to positively regulate the accumulation of RNA (78).

Thus, ever more experimental data regarding virus–host interactions are currently being generated. In an attempt to provide some clarity with respect to the complex analysis of the significance of the data, Li and coworkers generated an approach that defines potential regulatory networks of viral proteins, human miRNAs, and putative miRNA transcription factors between host targets (79).

## Deregulation of miRNAs in Cancer with Implications for Viral Activity

As already mentioned, miRNA signatures can not only distinguish between normal and cancer cells but also between cancer subtypes, and even between the cell types conforming the tumor itself. Studies have shown that lower expression, or even loss of miRNAs, is commonly found in tumor cells (80, 81), where most of them are recognized as tumor suppressors. On the other hand, fewer miRNAs are overexpressed in cancer cells and are considered oncomiRs, since they tend to be involved in tumorigenic processes. Both oncomiRs and tumor suppressor miRNAs contribute to different stages of carcinogenesis (82). On this basis, attempts to modulate miRNA expression are an important area of therapeutic development (83). Since many miRNAs are involved in tumorigenesis, the action of expressing or interfering with a single miRNA may have limited anti-cancer effects. The combination of multiple miRNAs with complementary mechanisms may impact on several signal transduction pathways, leading to an improved outcome. In this respect, multiple long non-coding RNAs have been designed for an adenovirus, aiming to cause it to bind to oncomiRs, instead of otherwise binding to endogenous targets, and thus achieving the interference of multiple miRNAs (84).

Cancer cells are coupled with abnormal signaling pathways and this has consequences for viral replication. For example, adenoviruses use interferon signaling to inhibit lytic virus replication in normal cells. However, they fail to inhibit it from replicating in cancer cells (85). The loss of interferon defenses in tumor cells is one of the mechanisms involved in the cancer selectivity of reovirus (86). Exploiting interferon deregulation in cancer is also a strategy employed to provide oncoselectivity for complex viruses (87, 88). Alterations to a variety of other pathways in cancer have constituted the principle option when seeking to confer cancer selectivity to viruses, with a view to cancer treatment (89, 90). Thus, although very little is yet known, one could speculate that the dysregulation of miRNAs in cancer may impact viral activity in tumor cells. Although the simplest rationale could claim that the more dysregulated miRNAs would be the first candidates when seeking to influence viral replication, recent observation illustrates that this might not always be the case and, in fact, functional interrogation would always be required. This is a point that was raised by the studies of Hodzic and coworkers, in which they showed that miR-26b, an abundant miRNA in prostate cancer cells, promoted adenovirus propagation and spread, leading to increased cell death (55). Further studies in this direction will provide a clearer view of the relevance that miRNA dysregulation in tumor cells may have with respect to modulating viral activity. Such a body of knowledge could constitute a novel platform in our quest to optimize oncolytic virotherapy.

## Concluding Remarks

Investigation of miRNAs has strongly impacted the field of oncolytic virotherapy. Many studies have shown their potential in precisely detargeting viral protein expression. The expression of viral protein in normal tissue is an undesired effect. They are highly immunogenic proteins that the body tends to eliminate, and can cause inflammation and cell death. Thus, the incorporation of MREs to regulate viral proteins has been key to improving the safety profile and therapeutic index of oncolytic virotherapy. Fine-tuning the design of the MRE has improved the efficacy of both cleavage and detargeting effects.

On the other hand, our understanding of the importance of the role of miRNAs in viral infections is increasing. Virus–host cell interaction impacts cellular miRNAs and alters their miRNome. Viruses take advantage of host cell miRNAs to promote virus replication, but cells react to viral infections by upregulating antiviral miRNAs. Interestingly, biological responses to the viral infection of cancer cells with abnormal signaling pathways are not the same as they would be with normal cells, and miRNAs would also seem to play a role in this differential response.

Up until now, much progress has been made in the engineering of oncolytic viruses with MREs in the attempt to provide improved selectivity and safety for their use. Future research may concentrate on further understanding the relationship between host miRNAs and viral replication, and how this may differentially impact normal and cancer cells. Such knowledge could prove fundamental and serve as the basis for exploiting newly engineered oncolytic viruses with enhanced antitumor potency.

## Author Contributions

CF coordinated the study and wrote some parts. XB-DR and MR-R wrote part of the manuscript.

## Conflict of Interest Statement

The authors declare that the research was conducted in the absence of any commercial or financial relationships that could be construed as a potential conflict of interest.
